# Patient characteristics associated with symptoms of anxiety, depression, and reduced body appreciation in women with polycystic ovary syndrome

**DOI:** 10.1093/humrep/deaf214

**Published:** 2025-11-03

**Authors:** T I Jannink, E M Bordewijk, V Lehmann, A Hoek, M Goddijn, M van Wely

**Affiliations:** Department of Obstetrics and Gynaecology, Centre for Reproductive Medicine, Amsterdam UMC, University of Amsterdam, Amsterdam, The Netherlands; Amsterdam Reproduction and Development Institute, Amsterdam UMC, Amsterdam, The Netherlands; Department of Obstetrics and Gynaecology, Centre for Reproductive Medicine, Amsterdam UMC, University of Amsterdam, Amsterdam, The Netherlands; Amsterdam Reproduction and Development Institute, Amsterdam UMC, Amsterdam, The Netherlands; Amsterdam Reproduction and Development Institute, Amsterdam UMC, Amsterdam, The Netherlands; Department of Medical Psychology, Amsterdam UMC, University of Amsterdam, Amsterdam, The Netherlands; Department of Obstetrics and Gynaecology, University of Groningen, University Medical Centre Groningen, Groningen, The Netherlands; Department of Obstetrics and Gynaecology, Centre for Reproductive Medicine, Amsterdam UMC, University of Amsterdam, Amsterdam, The Netherlands; Amsterdam Reproduction and Development Institute, Amsterdam UMC, Amsterdam, The Netherlands; Department of Obstetrics and Gynaecology, Centre for Reproductive Medicine, Amsterdam UMC, University of Amsterdam, Amsterdam, The Netherlands; Amsterdam Reproduction and Development Institute, Amsterdam UMC, Amsterdam, The Netherlands

**Keywords:** PCOS, anxiety, depression, body image, predictors

## Abstract

**STUDY QUESTION:**

Which polycystic ovary syndrome (PCOS)-related and general patient characteristics are associated with higher levels of anxiety and depressive symptoms, as well as with reduced body appreciation in women with PCOS?

**SUMMARY ANSWER:**

Anxiety was more common among participants with alopecia, obesity, younger age, and a history of anxiety or depression; depression was more common in participants with alopecia, unemployment, and a history of depression; and body appreciation scores were lower in participants with hirsutism, acne, alopecia, obesity, younger age, and a history of anxiety or depression.

**WHAT IS ALREADY KNOWN:**

Women diagnosed with PCOS face over 30% likelihood of clinically relevant anxiety symptoms, over a 15% likelihood of clinically relevant depressive symptoms, and also experience reduced body appreciation. Evidence suggests that in women with PCOS, various factors may contribute to increased levels of anxiety and depression and reduced body appreciation. However, findings across studies are inconsistent, and the nature of these associations, as well as the potential influence of patient characteristics that have been less studied, are still not well understood.

**STUDY DESIGN, SIZE, DURATION:**

A cross-sectional online survey study was carried out from May 2021 to July 2023. Recruitment occurred through fertility clinics in the Netherlands, employing posters, leaflets with QR codes, and online platforms run by patient organizations.

**PARTICIPANTS/MATERIALS, SETTING, METHODS:**

The participants were women with self-reported PCOS. They completed the Hospital Anxiety and Depression Scale (HADS) and the Body Appreciation Scale-2 (BAS-2). We assessed the association with mental health outcomes (symptoms of anxiety and depression, as well as body appreciation) with PCOS-related patient characteristics (hirsutism, acne, alopecia, obesity, and oligomenorrhea) and general characteristics (age, employment status, medical history, and medication use). Multivariable logistic and linear regression analyses were used, and adjusted odds ratios (aORs) or adjusted mean differences (aMDs) with 95% CI were calculated.

**MAIN RESULTS AND THE ROLE OF CHANCE:**

We included 982 women, with 37.0% showing clinically relevant symptoms of anxiety (score ≥11) and 17.4% showing clinically relevant depressive symptoms (score ≥11). Risk factors associated with anxiety symptoms were alopecia (aOR: 1.79, 95% CI 1.35–2.38), obesity (aOR: 1.40, 95% CI 1.03–1.90), younger age (aOR per year older: 0.93, 95% CI: 0.91–0.96), and medical history of anxiety or depression (aOR: 2.63, 95% CI 1.82–3.79 and aOR: 1.60, 95% CI 1.13–2.28). Risk factors associated with symptoms of depression were alopecia (aOR: 1.74, 95% CI 1.21–2.50), unemployment (aOR: 2.59, 95% CI 1.56–4.31), and a medical history of depression (aOR: 1.89, 95% CI 1.25–2.85). Risk factors associated with reduced body appreciation were hirsutism (aMD: −2.29, 95% CI −3.41 to −1.16), acne (aMD: −1.14, 95% CI −2.11 to −0.17), alopecia (aMD: −1.93, 95% CI −2.89 to −0.97), obesity (aMD: −6.31, 95% CI −7.36 to −5.27), oligomenorrhea (aMD: −1.81, 95% CI −2.78 to −0.83), and younger age (aMD per year older: 0.13, 95% CI 0.04–0.23). A medical history of anxiety or depression disorder was also associated with reduced body appreciation (aMD: −1.80, 95% CI −3.10 to −0.50; aMD: −2.81, 95% CI −4.05 to −1.57, respectively).

**LIMITATIONS, REASONS FOR CAUTION:**

Results are based on self-reported PCOS diagnoses and may have been affected by sampling bias.

**WIDER IMPLICATIONS OF THE FINDINGS:**

It is crucial for healthcare providers to understand which characteristics in women with PCOS may influence the development of anxiety, depression, or reduced body appreciation. Such awareness helps them to be more alert and better recognize the different types of mental health concerns, enabling referrals and more targeted mental health support.

**STUDY FUNDING/COMPETING INTEREST(S):**

This study was not funded by a specific grant. No conflicts of interest were reported in relation to the current research.

**TRIAL REGISTRATION NUMBER:**

Not applicable.

## Introduction

Polycystic ovary syndrome (PCOS) is a common hormonal disorder affecting between 6% and 13% of women of reproductive age (Bozdag *et al.*, 2016). This condition is diagnosed based on the Rotterdam Criteria, which include oligomenorrhea or amenorrhea, clinical or biochemical hyperandrogenism, and the presence of polycystic ovaries identified through ultrasound (Teede *et al.*, 2023; Balen *et al.*, 2024). Women with PCOS have an increased likelihood of being overweight or obese and may also face infertility due to anovulation (ESHRE/ASRM, 2004; Lim *et al.*, 2012). Additionally, women with PCOS often experience physical features of hyperandrogenism, such as hirsutism, acne, and alopecia, while they may also encounter health problems like insulin resistance, type 2 diabetes, and an increased risk of cardiovascular disease (Azziz *et al.*, 2004; Kakoly *et al.*, 2018; Zhang *et al.*, 2020).

Previous studies suggest that women with PCOS have a 30% increased likelihood of experiencing clinically relevant symptoms of anxiety and over a 15% likelihood of experiencing clinically relevant symptoms of depression (Cooney *et al.*, 2017; Jannink *et al.*, 2024). Additionally, women with PCOS have been found to report lower levels of body appreciation compared to women without the condition (Davitadze *et al.*, 2023).

Previous research suggests that clinical PCOS features, such as hirsutism, acne, alopecia, or obesity, may contribute to such increased levels of anxiety and depression and reduced body appreciation (Rasgon *et al.*, 2003; Himelein and Thatcher, 2006; Hollinrake *et al.*, 2007; Benson *et al.*, 2009; de Niet *et al.*, 2010; Asik *et al.*, 2015). However, findings across studies are inconsistent, with some reporting significant associations and others finding no such links (Bhattacharya and Jha, 2010). Importantly, most of these studies were limited by small sample sizes, assessed only a single mental health outcome, evaluated only a few potential risk factors, and often relied on average scores without adjusting for multiple variables. Furthermore, the impact of many other potentially relevant factors, like oligomenorrhea, age, medication use, and employment status, has been overlooked. Notably, employment status has been shown to be an important contributor to mental health outcomes in the general population (Franke *et al.*, 2024), yet its role in the mental health of women with PCOS remains unclear.

The current PCOS guidelines emphasize the importance of addressing mental health issues in women diagnosed with PCOS (Teede *et al.*, 2023). Thus, it is important to identify which women with PCOS are at increased risk of developing mental health issues. Such knowledge could support early detection and facilitate referrals to mental health services to improve the quality of life of women with PCOS in the long run.

Therefore, the aim of the present study is to investigate a range of PCOS-related features and general patient characteristics of women with PCOS and their relation to clinically relevant symptoms of anxiety and depression, as well as to reduced body appreciation.

## Material and methods

### Study design and participants

This is a web-based cross-sectional survey study, conducted as an extension of the ANxiety and DEpression Symptoms (ANDES) study (Jannink *et al.*, 2024), which focused on women with PCOS and women with other fertility problems. The ANDES study assessed the prevalence of mental health issues in infertile women undergoing fertility treatment, both with and without PCOS. In the present ANDES-II study, we focused on possible explanatory variables associated with symptoms of anxiety, depression, and body image issues among women with PCOS, regardless of whether they were undergoing fertility treatment.

Participants were recruited through two channels: first, two Dutch patient organizations (specifically ‘Stichting PCOS’ for women with PCOS and ‘Freya’ for couples with fertility problems) and 28 Dutch fertility clinics, which supported recruitment by displaying posters and distributing leaflets. The web-based questionnaire was accessible via internet links or QR codes. Data collection occurred between May 2021 until July 2023.

The Medical Ethics Committee of the Amsterdam UMC, location AMC, determined that the Medical Research Involving Human Subjects Act (WMO) did not apply to this study. The study was registered in the OSF Register under DOI 10.17605/OSF.IO/QBEAV.

### Inclusion and exclusion criteria

Women were included in the analyses if they were aged 18 years or older and within their reproductive years (i.e. pre-menopausal), since menopause can independently affect anxiety, depression, and other mental health outcomes in both women with PCOS and those without (Millán-de-Meer *et al.*, 2023). Women were also required to confirm in the online questionnaire that they had a medically verified diagnosis of PCOS. Women were excluded if they were pregnant, peri- or post-menopausal (including premature ovarian insufficiency) or had no ovaries at the time of this study.

### Measurements

The web-based questionnaire collected self-reported data on various demographic characteristics, including age, height, weight, and employment status. Additionally, the questionnaire gathered information on the presence of clinical hyperandrogenism symptoms, which included four categories: facial hirsutism, body hirsutism, acne, and alopecia. It did not record the specific locations of these symptoms. Questions about the menstrual cycle included cycle regularity, number of menstrual periods per year, and contraceptive use. The survey also assessed co-morbid conditions such as a diagnostic history of diabetes, eating disorders, anxiety, and depression, as well as antidepressant use. To assess whether participants met the PCOS diagnosis according to the Rotterdam criteria and fulfilled the inclusion and exclusion criteria, the survey evaluated the presence of ovaries and peri- or post-menopausal status.

Subsequently, participants were asked to complete two validated self-report questionnaires: the Dutch version of the Hospital Anxiety and Depression Scale (HADS) (Zigmond and Snaith, 1983) and the Body Appreciation Scale-2 (BAS-2) (Tylka and Wood-Barcalow, 2015). The HADS is a validated 14-item questionnaire with seven questions assessing anxiety symptoms (HADS-A subscale) and seven assessing depressive symptoms (HADS-D subscale). Each question is scored on a scale from 0 to 3, resulting in a total score ranging from 0 to 21 for both subscales. Higher scores correspond to increased levels of anxiety or depression, categorized as: no symptoms (scores 0–7), potential presence of anxiety or depression (8–10), or suspected manifestation of anxiety or depression disorder (11–21) (Herrmann, 1997). The 10-item BAS-2 assesses body appreciation in terms of body acceptance, body respect and care, as well as resistance to media-promoted appearance ideals. All items are rated on a 5-point scale ranging from 1 (*never*) to 5 (*always*) and summed up to one total score (possible range: 10–50), with higher scores indicating more positive body appreciation.

### Outcomes

Primary outcomes are clinically significant symptoms of anxiety or depression, determined as a score of ≥11 (Benson *et al.*, 2009; Cinar *et al.*, 2011), as well as mean BAS-2 scores, reflecting participants’ body appreciation. Secondary outcomes include the proportion of participants with anxiety and depression scores of 8 or higher (i.e. potential anxiety or depression) and mean anxiety and depression scores. These secondary outcomes allow for comparison to other research using similar cutoffs.

### Potential risk factors

We included the following PCOS-related characteristics (categorized as ‘present’ or ‘absent’): facial hirsutism, body hirsutism, acne, alopecia, obesity (BMI ≥30), and oligomenorrhea (defined as having fewer than 8 menstrual cycles per year; Teede *et al.*, 2023). We included the following general patient characteristics: age (continuous variable), employment status, medical history of anxiety, depression or eating disorder, and antidepressant or contraceptive use (categorized as ‘yes’ or ‘no’).

### Data handling

The study data were collected using the LimeSurvey web-based questionnaire tool, which ensures the confidential processing of all data. After data collection was finished, the data were transferred to an SPSS file and saved on secure servers at the Amsterdam UMC.

### Statistical analysis

Analyses were performed using the IBM SPSS software (version 28.0; IBM Corp., USA). A two-sided *P*-value of <0.05 was considered statistically significant.

Continuous data were assessed for normality and expressed as mean ± SD; categorical data were expressed as proportions.

Baseline characteristics were determined for all participants and reported using descriptive statistics. Prevalence and mean values of mental health outcomes (anxiety symptoms, depression symptoms, and body appreciation) were computed.

The potential risk factors were checked for multicollinearity by preparing bivariate correlation matrix coefficients of all pairs of independent variables. We selected the variable oligomenorrhea (less than 8 menstrual periods per year) for our model instead of irregular cycles, as we believed this provides a more reliable reflection of the participant’s current menstrual pattern.

We used multivariate binary logistic regression to assess the association between PCOS-related and general characteristics with mental health outcomes (i.e. clinically relevant anxiety and depression). Associations were expressed in adjusted odds ratios (aORs) with corresponding 95% CI. We used multivariate generalized linear regression to determine the association between patient characteristics and body appreciation scores, with associations expressed in adjusted mean differences (aMDs) with 95% CI. All estimates include 95% CIs based on 1000 bootstrap samples.

## Results

Out of all women who volunteered to complete our online survey (n = 1038), we included 982 (95%) eligible women for this study. We excluded 56 women because they were menopausal (n = 22) or currently pregnant (n = 34).

### Patient characteristics

The participants had an average age of 30.5 years (±5.2) and an average BMI of 27.5 (±6.3), with 58.2% being considered overweight (i.e. BMI ≥25 kg/m^2^) and 32.6% being obese (i.e. BMI ≥30 kg/m^2^; Table 1). Unemployment was reported by 8.8% of the participants. The majority of women (73.3%) had polycystic ovaries. Facial hirsutism was present in 66.4% of the women and body hirsutism in 58.6%, with 50.8% reporting both symptoms. Additionally, acne was present in 51.0% and alopecia in 51.3% of participants. Any clinical sign of hyperandrogenism was found in 94.5% of participants. Irregular menstrual cycles were reported by 80.9% of participants, with 56.2% having less than 8 cycles per year (in this study defined as having oligomenorrhea). The average number of menstrual cycles in the past year was 7.4 (±4.0).

**Table 1.** Descriptive statistics of participants.

	PCOS (N = 982)
Age of women (years)	30.5 ± 5.2
BMI (kg/m^2^)	27.5 ± 6.3
Overweight (≥25 kg/m^2^)	572 (58.2%)
Obese (≥30 kg/m^2^)	320 (32.6%)
*Education*	
Primary and secondary education	80 (8.1%)
Secondary vocational education	324 (33.0%)
Higher professional education	352 (35.8%)
University education	226 (23.0%)
Unemployment	86 (8.8%)
Polycystic ovaries	720 (73.3%)
Any clinical signs of hyperandrogenism	928 (94.5%)
*Categories*	
Facial hirsutism	652 (66.4%)
Body hirsutism	575 (58.6%)
Acne	501 (51.0%)
Alopecia	504 (51.3%)
*Menstrual cycle*	
Experiencing irregular cycles	794 (80.9%)
Oligomenorrhea (<8 cycles/year)	552 (56.2%)
Mean number of periods in the past 12 months	7.4 ± 4.0
Use of contraceptives	227 (23.1%)
*Mental health history*	
Anxiety disorder	188 (19.1%)
Depression disorder	237 (24.1%)
Eating disorder	61 (6.2%)
Use of antidepressants	37 (3.8%)
*Complete data*	
HADS	982 (100%)
BAS-2	964 (98.2%)

Data are N (%) or mean ± SD. HADS: Hospital Anxiety and Depression Scale; BAS-2: Body Appreciation Scale-2; PCOS: polycystic ovary syndrome.

A medical history of a diagnosed anxiety disorder was reported by 19.1% of all participants, a medical history of a depression disorder was reported by 24.1%, and a medical history of an eating disorder was reported by 6.2%. Furthermore, 3.8% of participants used antidepressants at the time of study. Additional descriptive statistics can be found in Table 1.

### Prevalence of anxiety, depression, and body appreciation

Clinically relevant levels of anxiety symptoms (HADS-A scores ≥11) were found in 37.0% of women (Table 2). Additionally, 59.5% of women had HADS-A scores of 8 or higher (i.e. potential anxiety). Clinically relevant levels of depressive symptoms (HADS-D scores ≥11) were observed in 17.4% of the women, with 36.4% having HADS-D scores of 8 or above (i.e. potential depression).

**Table 2.** HADS-anxiety, HADS-depression, and BAS-2 scores.

	PCOS (N = 982)
**Primary outcomes**	
HADS-A score ≥ 11	363 (37.0%)
HADS-D score ≥ 11	171 (17.4%)
BAS-2 mean score	31.6 ± 8.6
**Secondary outcomes**	
HADS-A score ≥ 8	584 (59.5%)
HADS-A mean score	9.0 ± 4.5
HADS-D score ≥ 8	357 (36.4%)
HADS-D mean score	6.4 ± 4.1

Data are N (%) or mean ± SD. HADS-A: Hospital Anxiety and Depression Scale: anxiety score; HADS-D: Hospital Anxiety and Depression Scale: depression score; BAS-2: Body Appreciation Scale-2; PCOS: polycystic ovary syndrome.

The average summed body appreciation score (BAS-2 score) was 31.6 (±8.6), with a range from 10 to 50 (Table 2).

### Associations between patient characteristics and mental health outcomes

#### Anxiety

**Figure 1.**
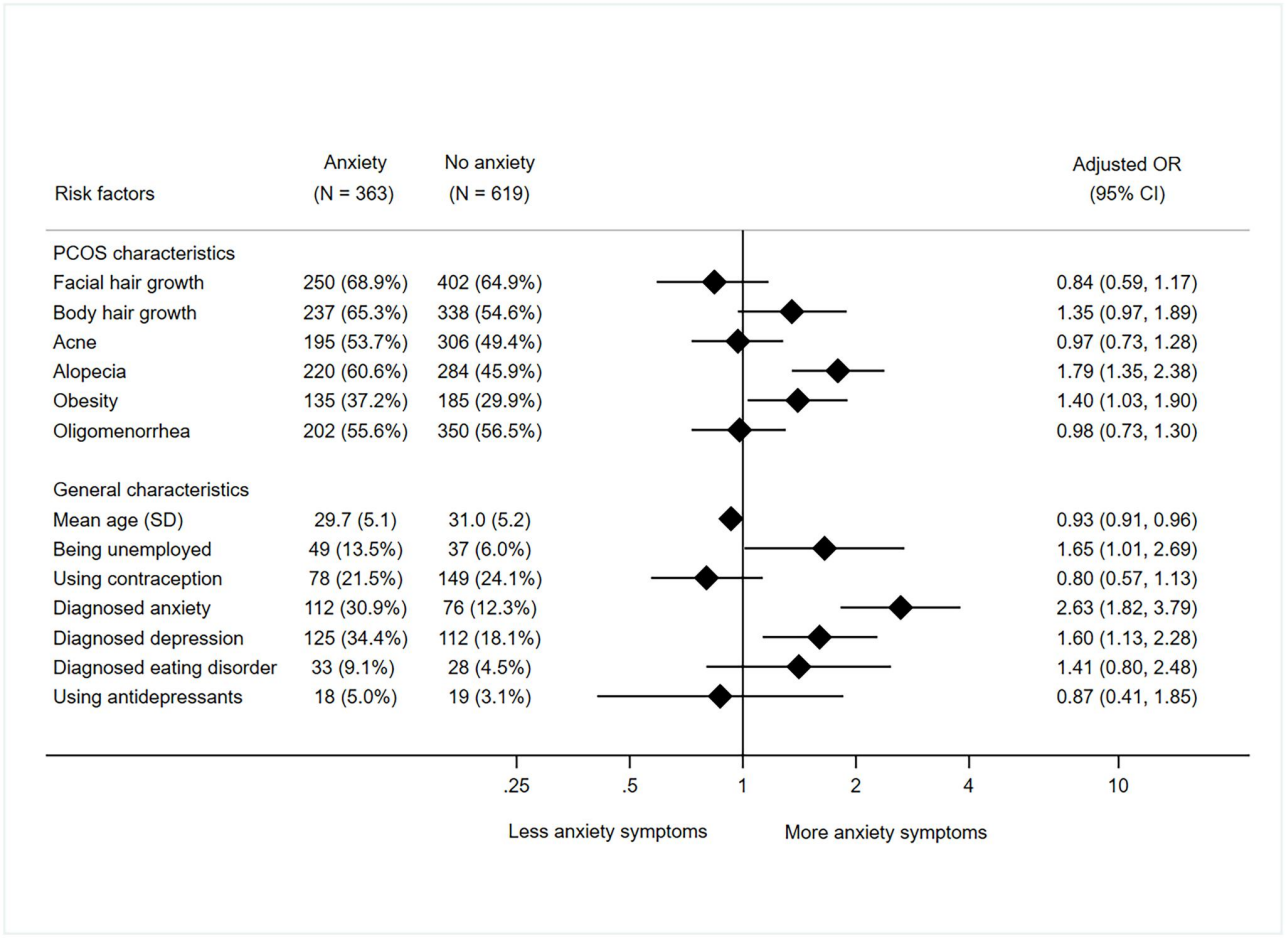
**Associations between patient characteristics and clinically relevant anxiety symptoms (HADS-A ≥ 11).** Presence of a risk factor in women with or without clinically relevant anxiety is expressed as N (%). Oligomenorrhea: defined as having fewer than 8 menstrual cycles per year. HADS-A: Hospital Anxiety and Depression scale: anxiety score; OR: odds ratio.

Of all signs of hyperandrogenism, only alopecia was significantly associated with higher levels of clinically relevant anxiety (aOR: 1.79, 95% CI 1.35–2.38; Fig. 1). Other factors that were significantly associated with increased anxiety symptoms were obesity (aOR: 1.40, 95% CI 1.03–1.90), younger age (aOR per year older: 0.93, 95% CI: 0.91–0.96), and a medical history of anxiety (aOR: 2.63, 95% CI 1.82–3.79) or depression (aOR: 1.60, 95% CI 1.13–2.28).

#### Depression

**Figure 2.**
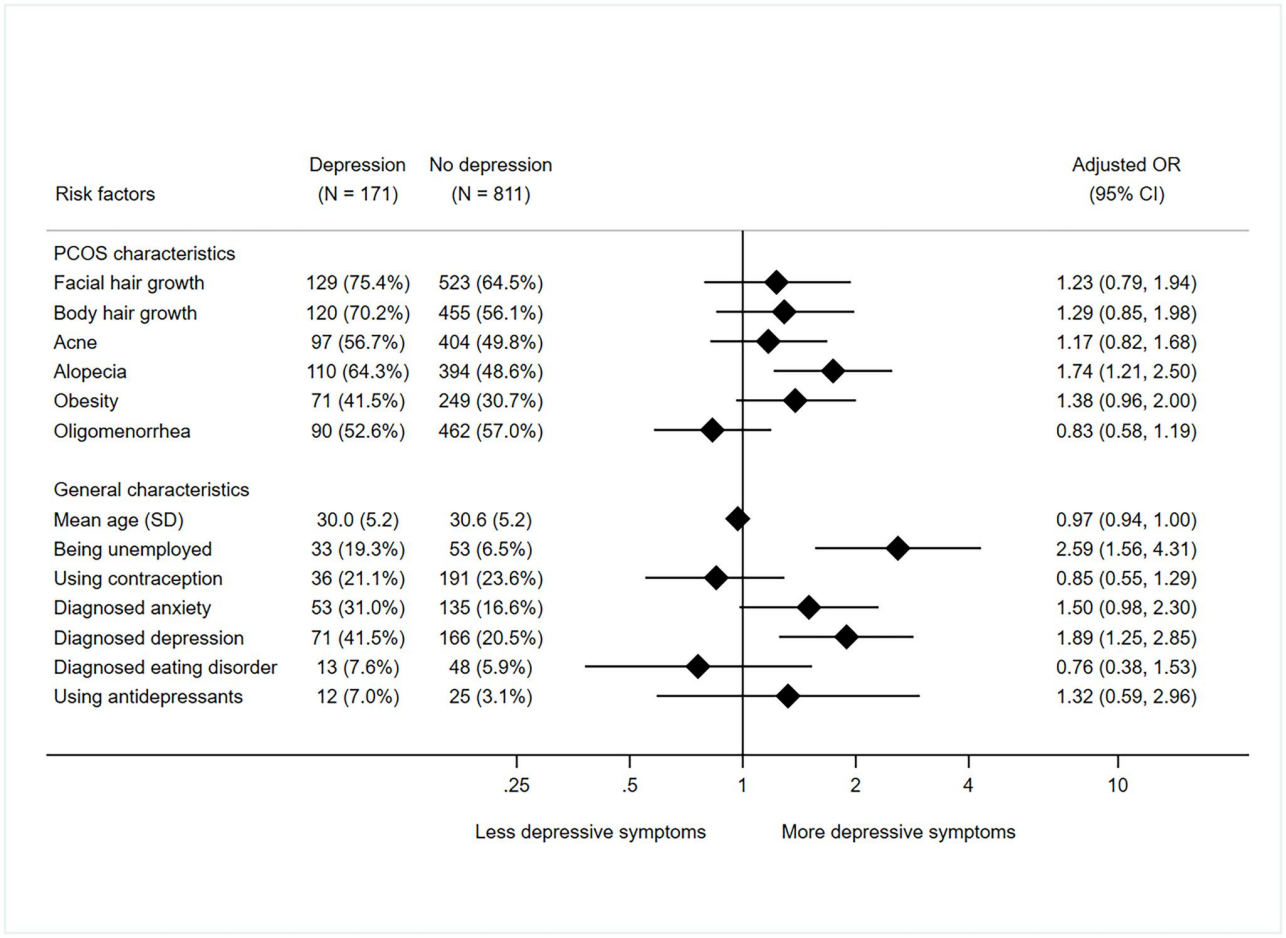
**Associations between patient characteristics and clinically relevant depressive symptoms (HADS-D ≥ 11).** Presence of a risk factor in women with or without clinically relevant depression is expressed as N (%). Oligomenorrhea: defined as having fewer than 8 menstrual cycles per year. HADS-D: Hospital Anxiety and Depression scale: depression score; OR: odds ratio.

Likewise, among the clinical signs of hyperandrogenism, only alopecia was associated with higher levels of clinically relevant depression symptoms (aOR: 1.74, 95% CI 1.21–2.50; Fig. 2). Higher levels of depression were observed in unemployed participants (aOR: 2.59, 95% CI 1.56–4.31) and those who had a medical history of depression (aOR: 1.89, 95% CI 1.25–2.85).

#### Body appreciation

**Figure 3.**
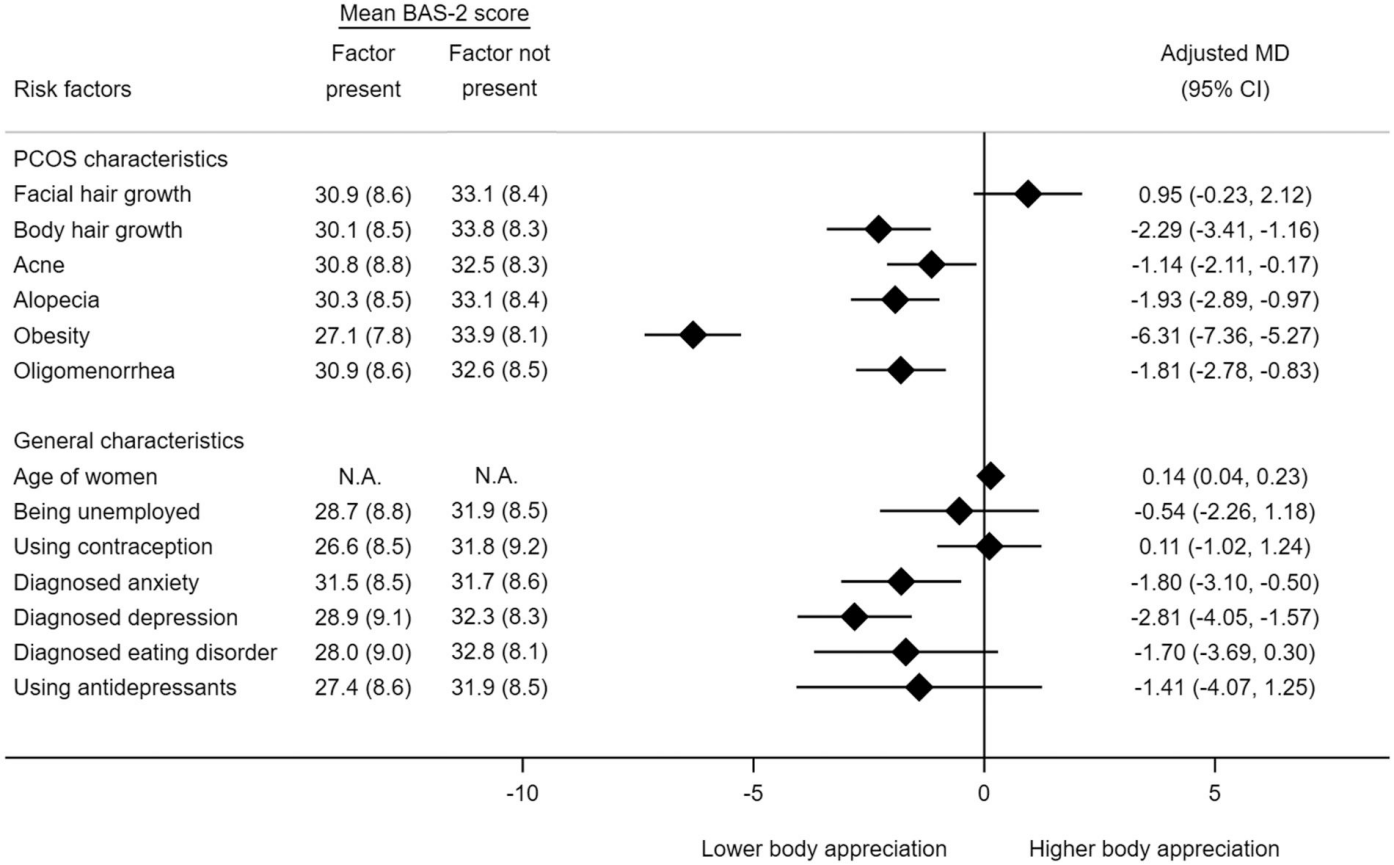
**Associations between patient characteristics and body appreciation scores (mean BAS-2).** Mean BAS-2 scores with SDs are presented by the presence or absence of a risk factor. Oligomenorrhea: defined as having fewer than 8 menstrual cycles per year. BAS-2: Body Appreciation Scale-2; MD: mean difference.

Hirsutism (body hair growth), acne, and alopecia were all associated with reduced body appreciation scores (aMD: −2.29, 95% CI −3.41 to −1.16; aMD: −1.14, 95% CI −2.11 to −0.17; aMD: −1.93, 95% CI −2.89 to −0.97; Fig. 3). Additionally, a strong association was found between obesity and reduced body appreciation (aMD: −6.31, 95% CI −7.36 to −5.27). Furthermore, body appreciation was reduced in participants with oligomenorrhea (aMD: −1.81, 95% CI −2.78 to −0.83) and in younger participants (aMD per year older: 0.13, 95% CI 0.04–0.23). Body appreciation scores were also found to be lower in women with a medical history of anxiety or depression relative to women without such history (aMD: −1.80, 95% CI −3.10 to −0.50 and aMD: −2.81, 95% CI −4.05 to −1.57, respectively).

## Discussion

Our study showed a prevalence of clinically relevant anxiety and/or depressive symptoms of 37.0% and 17.4%, respectively, which is consistent with previous research (Benson *et al.*, 2009; Barry *et al.*, 2011). Also, averaged BAS-2 scores were comparable to those scores previously found in women with PCOS (Geller *et al.*, 2025). More importantly, we found that several PCOS-related and general patient characteristics are associated with clinically relevant symptoms of anxiety and depression, as well as with reduced body appreciation.

Of all tested clinical signs of hyperandrogenism, alopecia emerged as a significant predictor of all investigated mental health outcomes. This finding aligns with a recent study linking alopecia to body image concerns in women with PCOS (Gao *et al.*, 2025). To the best of our knowledge, only one small study has specifically examined the relationship of alopecia with anxiety and depression in this population, reporting a significant association with anxiety, but not with depression (Chaudhari *et al.*, 2018). Yet, in other patient populations, female alopecia has been associated with poorer mental health and reduced quality of life (Davis and Callender, 2018; Hwang *et al.*, 2024), further supporting our findings that alopecia can be relevant for both anxiety and depression. Given that half of our participants reported alopecia, we also emphasize the need for further research to better understand its impact among women with PCOS.

Additionally, hirsutism, acne, and oligomenorrhea were significantly associated with lower body appreciation, consistent with previous literature (de Niet *et al.*, 2010; Pastore *et al.*, 2011; Bazarganipour *et al.*, 2013). Interestingly, we did not observe associations between these PCOS-related symptoms and anxiety or depression, which contrasts with previous research. Indeed, other studies have reported associations between hirsutism and anxiety (Asik *et al.*, 2015), acne and anxiety (Benson *et al.*, 2009), and menstrual irregularities and depression (Bazarganipour *et al.*, 2013). Our findings suggest that, in this population, the visible and clinical manifestations of hyperandrogenism may influence body image more consistently than they influence psychological distress, such as anxiety or depression. However, caution is needed in clinical care given that symptoms of anxiety and depression can be rather strongly related to body appreciation (Jannink *et al.*, 2024).

In our study, we differentiated between facial hair and body hair growth to gain a more detailed understanding of the psychological impact of hirsutism. Notably, we did not find a significant association between facial hair growth and psychological outcomes, despite previous research highlighting the burden of facial hair in the general population (Lipton *et al.*, 2006). One possible interpretation is that facial hair might be more easily managed through methods such as epilation or laser treatment. However, this remains speculative and warrants further investigation.

We found significant associations between obesity and both anxiety and reduced body appreciation. These findings align with recent literature showing that higher BMI is associated with poorer body image and elevated levels of anxiety (Hofmann *et al.*, 2025). Although the difference in depressive symptoms was not statistically significant in our sample, we observed a trend suggesting that women with obesity were more likely to experience clinically relevant levels of depression, consistent with previous research (Benson *et al.*, 2009; Cinar *et al.*, 2011; Cooney *et al.*, 2017). Such association may be influenced by factors such as weight-related stigma, body dissatisfaction, and internalized shame (Puhl and Heuer, 2009). Given that one-third of our sample was classified as obese, awareness for obesity as a predictor of psychological vulnerability in women with PCOS is important.

Regarding general characteristics, we found that younger age emerged as a risk factor for anxiety symptoms and reduced body appreciation. This association has not been reported in other studies on women with PCOS (Bazarganipour *et al.*, 2013; Lin *et al.*, 2021), although it has been observed in the general population (Christensen *et al.*, 1999), possibly reflecting that older individuals may have developed more effective coping strategies and greater emotion regulation abilities (Gross *et al.*, 1997). The relationship between younger age and reduced body appreciation has been described earlier in the general population and may be explained by women’s increasing appreciation of their health and greater acceptance of their bodies over time (Tiggemann and McCourt, 2013). Furthermore, unemployment was associated with depressive symptoms, consistent with prior findings in the general population (Paul and Moser, 2009; Zuelke *et al.*, 2018), which might be explained by several factors, including financial stress, reduced social interaction, and lower self-esteem (Butterworth *et al.*, 2009; Paul and Moser, 2009).

The association between a medical history of depression and the mental health outcomes may be unsurprising, just as a history of anxiety was found to be a significant predictor of anxiety symptoms and reduced body appreciation. However, its clinical significance remains considerable. Anxiety and depression often co-occur (Kessler *et al.*, 2003), and previous research in women with PCOS has highlighted the interplay between various mental health outcomes and body image (Alur-Gupta *et al.*, 2019; Jannink *et al.*, 2024). Our findings suggest that a history of one mental health condition may influence the development or severity of another. Therefore, it remains important for clinicians to be aware of such associations and to ask patients about their mental health and psychiatric history, as they represent significant risk factors for current psychological distress.

Although we expected that a history of eating disorders might negatively affect body appreciation, this was not reflected in our results. While we cannot fully explain this finding, it may be possible that past treatments have played a role in mitigating the impact. Additionally, the use of antidepressants or contraceptives did not appear to affect our mental health outcomes, which may possibly suggest that other patient characteristics had a stronger influence, or that these medications helped stabilize mental health. However, as only 3% of participants reported antidepressant use, the subgroup may have been too small to detect a reliable association.

This study has several strengths. First, the large sample size from multiple clinics across the country enhances the national generalizability and statistical power of our findings.

Additionally, our study is novel in investigating the associations of various PCOS-related characteristics, including alopecia, as well as other potentially associated patient characteristics with multiple mental health outcomes.

By including a wide range of patient variables in multivariable analyses, we were able to adjust for potential confounders and check for interactions, thus improving the validity of our results. Furthermore, we distinguished between facial and body hair growth, providing a more detailed understanding of the psychological impact of hirsutism.

At the same time, the study has several limitations. Due to the design of this study, PCOS diagnoses were based on self-report without clinical confirmation or verification through medical records. This raises the possibility of diagnostic misclassification, which could influence the strength of observed associations. Additionally, the recruitment method of convenience sampling through patient organizations may have led to selection bias (e.g. women experiencing greater psychological distress may have been more likely to participate). Recruitment through fertility clinics may have further contributed to selection bias, as infertility itself can be a significant source of psychological burden, independent of PCOS status (Jannink *et al.*, 2024). Nevertheless, our identified prevalence of clinically relevant symptoms of anxiety and depression was comparable to other studies.

Moreover, our cross-sectional design limits causal inference, making it impossible to determine whether PCOS features contribute to the development of mental health symptoms or vice versa. Our findings are further limited by our measures to examine PCOS features (e.g. hirsutism and alopecia), for which we used simplified scoring methods (i.e. present/absent) instead of standardized scoring methods (e.g. the Ferriman-Gallwey scale and the Ludwig scale), which constrains interpretability. Furthermore, we could not account for additional potential confounders, such as cultural and socioeconomic factors, or the use of medications like metformin and anti-androgens, which may affect both PCOS symptoms and mental health. Finally, because the study population was drawn from a Dutch sample, the generalizability of our findings to more diverse cultural or healthcare contexts remains uncertain.

Despite these limitations, our findings have important clinical implications. Our study has provided more understanding into specific patient characteristics associated with the presence of anxiety and depressive symptoms, or reduced body appreciation, in women with PCOS. Furthermore, our findings highlight the impact of PCOS-related features, particularly the clinical manifestations of hyperandrogenism, on mental wellbeing. The observed associations can help healthcare providers to become more aware of potential mental health problems in certain women with PCOS and can contribute to earlier recognition of problems.

We encourage healthcare providers to address mental health directly in the consultation room and to offer referrals to mental health specialists when needed. Screening instruments (such as the Beck Anxiety Inventory (BAI) or Beck Depression Inventory-second Edition (BDI-2)) could support this process, but their routine use may also increase healthcare burden and potentially contribute to stigma or overdiagnosis given the moderate to high prevalence of anxiety and depression (Kitzinger and Willmott, 2002). Nevertheless, we believe that initiating dialogue and acknowledging mental health concerns can already play a meaningful role in improving care for women with PCOS, even in the context of limited resources.

For future research, we recommend longitudinal studies with clinically verified PCOS diagnoses and standardized assessment of PCOS features. Replicating similar research in other countries or cultural contexts is also vital to investigate possible differences in associations between patient characteristics and mental health problems across diverse populations. It is essential to provide appropriate care to women with PCOS worldwide.

## Data Availability

The data underlying this article will be shared upon reasonable request to the corresponding author.
